# Calcium Phosphate as a Key Material for Socially Responsible Tissue Engineering

**DOI:** 10.3390/ma9060434

**Published:** 2016-06-01

**Authors:** Vuk Uskoković, Victoria M. Wu

**Affiliations:** 1 Department of Bioengineering, University of Illinois, Chicago, IL 60607-7052, USA; victoriamwu1015@gmail.com; 2 Department of Biomedical and Pharmaceutical Sciences, Chapman University, Irvine, CA 92618-1908, USA

**Keywords:** antimicrobials, biomaterials, biomedicine, calcium phosphate, drug delivery, hydroxyapatite, nanomaterials, nanotechnology, social responsibility, sustainability

## Abstract

Socially responsible technologies are designed while taking into consideration the socioeconomic, geopolitical and environmental limitations of regions in which they will be implemented. In the medical context, this involves making therapeutic platforms more accessible and affordable to patients in poor regions of the world wherein a given disease is endemic. This often necessitates going against the reigning trend of making therapeutic nanoparticles ever more structurally complex and expensive. However, studies aimed at simplifying materials and formulations while maintaining the functionality and therapeutic response of their more complex counterparts seldom provoke a significant interest in the scientific community. In this review we demonstrate that such compositional simplifications are meaningful when it comes to the design of a solution for osteomyelitis, a disease that is in its natural, non-postoperative form particularly prevalent in the underdeveloped parts of the world wherein poverty, poor sanitary conditions, and chronically compromised defense lines of the immune system are the norm. We show that calcium phosphate nanoparticles, which are inexpensive to make, could be chemically designed to possess the same functionality as a hypothetic mixture additionally composed of: (a) a bone growth factor; (b) an antibiotic for prophylactic or anti-infective purposes; (c) a bisphosphonate as an antiresorptive compound; (d) a viral vector to enable the intracellular delivery of therapeutics; (e) a luminescent dye; (f) a radiographic component; (g) an imaging contrast agent; (h) a magnetic domain; and (i) polymers as viscous components enabling the injectability of the material and acting as carriers for the sustained release of a drug. In particular, calcium phosphates could: (a) produce tunable drug release profiles; (b) take the form of viscous and injectable, self-setting pastes; (c) be naturally osteo-inductive and inhibitory for osteoclastogenesis; (d) intracellularly deliver bioactive compounds; (e) accommodate an array of functional ions; (f) be processed into macroporous constructs for tissue engineering; and (g) be naturally antimicrobial. All in all, we see in calcium phosphates the presence of a protean nature whose therapeutic potentials have been barely tapped into.

## 1. Introduction

“I have to leave the convent and consecrate myself to the poor” [1].

Mother Teresa, Calcutta—Darjeeling train, 1946.

Socially responsible design of nanomaterials, including therapeutic nanostructures, is rarely addressed. Only one publication listed in Elsevier’s bibliographic database Scopus contains both the terms “socially responsible” and “nano” [2]. Socially responsible design is tied to the aim of acting for the benefit of society at large and oftentimes involves making the product affordable to impoverished populations through structural and compositional simplification. This approach, however, clashes with the common assumption that the more structurally and functionally complex the nanoparticle, the greater its applicative potentials. For this reason, structural and functional complexification of nanoparticles elicits a greater interest from the scientific community than their simplification. Quite often, however, an extensive cost is associated with synthesis and characterization (necessary to verify the structure and the correct composition) of such complex nanostructures. As such, their consideration for a prompt therapeutic application in poor regions of the world and underprivileged communities is equivocal, if not illusory.

**Figure 1:**
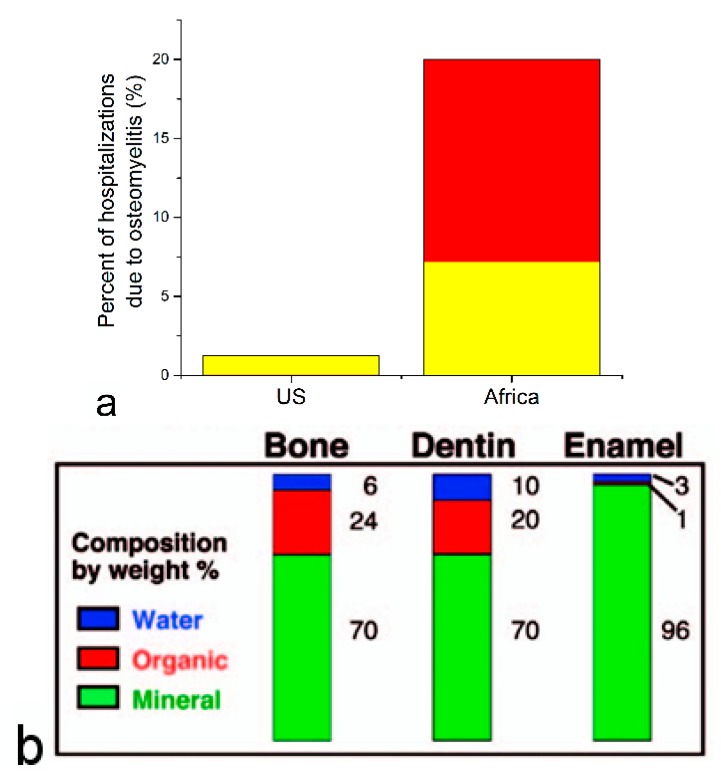
(**a**) Percentage of hospitalizations due to osteomyelitis, equaling 1.23% in the United States and ranging from 7% to 20% in Africa. The area below and above 7% differs in color—yellow and red, respectively. Data retrieved from Refs. [7,8,9]; (**b**) Percentage of mineral, organic, and aqueous components of three major hard tissues in the human body, demonstrating calcium phosphate (CAP) as the most abundant component of each of the three of them: bone, dentin and enamel. Adapted from [10] with permission from ©2008 Mineralogical Society of America.

One example comes from osteomyelitis, the disease that is prevalent in poor sanitary conditions typical for underdeveloped regions of the world [3]. Although an interest for finding more advanced and patient-friendly solutions to it has recently emerged because the disease is often secondary to bone surgeries [4,5], the frequency of which follows in step with the continuously aging average population of the developed world, in its natural form it is predominantly present in environments where low hygiene, malnutrition, other infectious diseases, compromised immunity and poverty are endemic. Figure 1a, for example, illustrates the disparity between the percentage of hospitalizations due to bone infection in the US and in Africa: 1%–2% *vs.* 7%–20%, respectively. In this non-postoperative form, the disease is, moreover, particularly known for striking children [6]. Ideally, therefore, the therapy for osteomyelitis should be affordable to the inhabitants of poorer regions of the world than those in which the most advanced tissue engineering solutions for bone disease are being developed today.

Typical therapies for osteomyelitis include oral or parenteral administrations of antibiotics ranging in duration from 2–6 weeks depending on the severity of infection as well as surgical debridement of the infected and necrotic tissue [11,12]. This rather invasive therapy suffers from numerous drawbacks, including (a) the side effects tied with systemic distribution of antibiotics; (b) their low bioavailability due to impaired vascularization of an already lowly vascularized boney tissues; (c) skeletal disfigurement resulting from the surgical intervention; and (d) the risk of new infection accompanying open surgery [13]. Therefore, attempted to be developed are therapies involving the minimally invasive injection of a colloidal carrier with (a) sustained antibiotic release profiles for local delivery; and (b) osteogenic properties so as to minimize bone loss and maximize its regeneration. The pivotal place in many of them is occupied by calcium phosphate (CAP), the major component of all hard tissues in the human body (Figure 1b).

**Figure 2:**
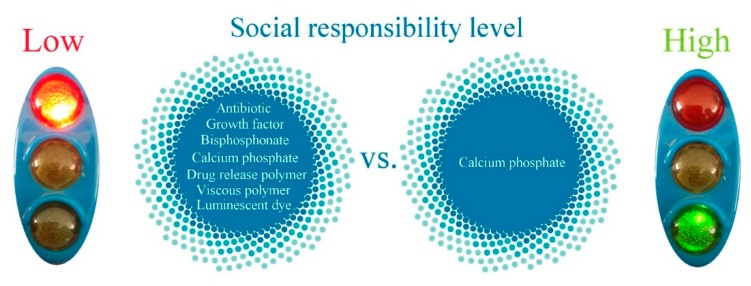
Two hypothetic bone graft formulations, one containing a multitude of functional components and the other one containing only calcium phosphate, albeit capable of possessing all the functional properties of the multicomponent formulation. Given their identical medical performance, the social responsibility levels of the two would differ: the first ones would be classified as low and the second ones as high.

CAP is a material capable of exhibiting an unusually wide variety of properties; it is this versatility of CAP that presents the basis for its consideration as a candidate for the key material for socially responsible tissue engineering. As an illustration of this point, Figure 2 depicts two hypothetic formulations. One of them contains (a) CAP as a central ingredient, *i.e.*, the filler, so to speak; (b) a bone growth factor, e.g., bone morphogenetic protein-2; (c) an antibiotic for prophylactic or anti-infective purposes; (d) a bisphosphonate as an antiresorptive compound; (e) a viral vector to enable the intracellular delivery of therapeutics; (f) a luminescent dye; (g) a radiographic component; (h) an imaging contrast agent; (i) a magnetic domain; (j) one polymer as a viscous component enabling the injectability of the material; and (k) another one acting to allow for the sustained release of a drug. The other one contains only CAP. If we were to ask the patients, the clinicians, the chemists, and the regulatory agencies which of these formulations would be preferred, there is no doubt that everybody would be in favor of the latter. The reasons are obvious: the compositional and synthetic simplicity, lower fabrication costs, greater availability of the reagents, higher technological transferability, *in situ* synthesizability and a lesser risk of side effects. Table 1, for example, illustrates how economical CAPs, at the price of 6–12 cents per gram, are compared to all the other commercially available components of advanced bone graft formulations, some of which exceed the cost of CAPs by ten orders of magnitude (e.g., the angiogenic growth factor TGF-β whose current price exceeds $200 million per gram). However, how could CAP alone possess all these properties imparted to a bone graft material by the aforementioned additives? In what follows we will try to convince the reader that the formulations are indeed possible wherein CAP *per se* would possess all the sundry of properties that an ideal bone graft material should possess. We will uncover some of the properties and functionalities displayable by CAP that prove the protean nature of this solid whose peculiarity is analogous to that of water in the realm of liquids [14]. Clinicians who have derogatorily compared the implantation of CAP to the implantation of “stones” [15] might be pleasantly surprised upon the realization of this myriad of exciting properties latently concealed in the structure of the seemingly unattractive and simple material that CAP is. The elucidation of this versatility of properties was presented in earlier review studies [16]; here it serves the sole role of supporting the reasons for which CAP could be seen as fundamental to the new generation of socially responsible materials for tissue engineering and other biomedical applications.

## 2. CAP as a Tunable Drug Release Carrier and a Viscous, Self-Setting Material for Injectable Bone Grafts

CAP has plenty of properties for which it is desired in bone graft formulations [17]. It is a bioactive, biocompatible, biodegradable, osteoconductive, non-immunogenic component of bone. If we were to take into account the ancient Latin maxim, *similia similibus curantur* (“like cures like”), it could indeed be considered as an ideal component of tissue engineering constructs for the replacement of bone. However, being a ceramic material, CAP is all but an ideal drug delivery carrier. Namely, the lattice formation energy is large enough to expel most organic molecules precipitated together with CAP. Therefore, unlike polymers, which could entrap drugs inside the particle, no organic molecule larger than glycine can be accommodated inside the crystal lattice of CAP through intercalation (even the entrapment of glycine inside hydroxyapatite is conditioned by defects in the lattice and the presence of paired OH^−^ and Ca^2+^ vacancies) [18]. On top of this, the presence of the hydrated and intensely mobile layer of ions, whose crystalline order, if any, has little in common with the bulk of the particle [19,20], allows for the rapid exchange of ions across the particle/solution interface and disables stable chemical bonding of organic molecules to the CAP particle surface. This inherently unstructured and mobile surface layer of CAP particles in solution, unlike that typifying most oxide ceramics, also allows for the characteristic, aggregational growth of CAP particles [21], whereby smaller, amorphous or crystalline units get “sintered” under ambient conditions thanks to the low energy barrier for the coalescence of their diffusive, highly hydrated surfaces. Such aggregational growth, in theory, makes the entrapment of organics within ultrafine CAP nanoparticle aggregates possible, though this is still a *terra incognita* in our understanding of CAP/organic interface. Nevertheless, drug loading via co-precipitation onto CAP at the atomic scale is limited to physisorption, given that there is no ability to entrap the drug within the crystal lattice or have it chemically conjugated to the surface. On one hand, owing to the alternation of intensely charged multivalent species (Ca^2+^, PO_4_^3−^) on the particle surface, CAP can relatively strongly bind a variety of organic molecules [22,23,24]. On the other hand, such strong binding usually does not stand in the way of a considerable amount of burst release that occurs in the first minutes of the contact of the material with the solution [25]. To promote sustained release of drugs from CAPs, such as that achievable using biodegradable polymers [26,27,28], thus stands as an enormous challenge.

**Figure 3:**
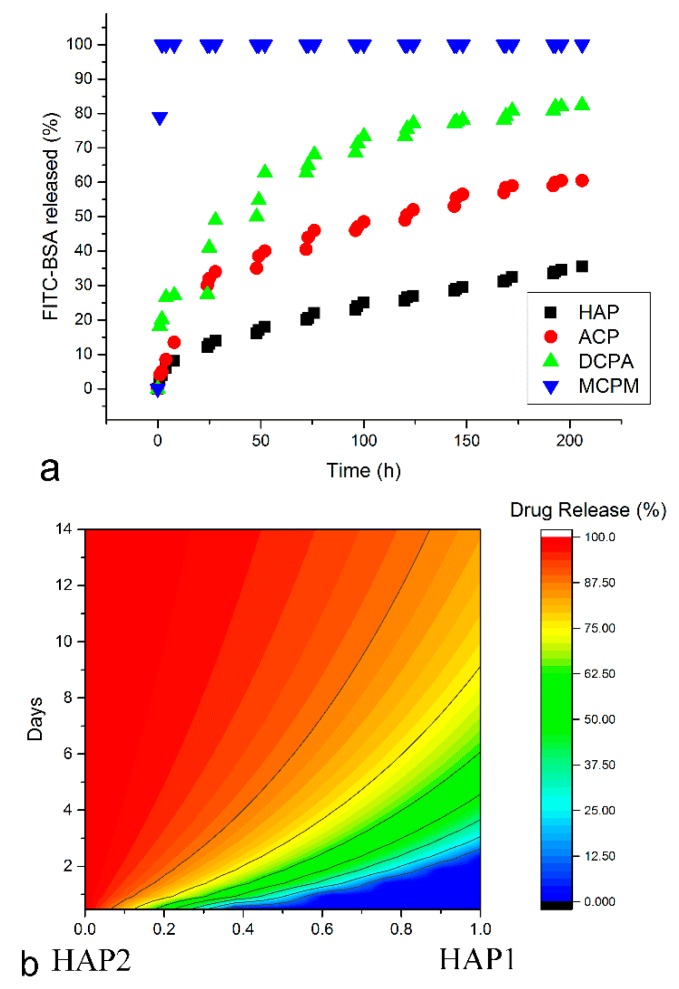
(**a**) Release of serum albumin (FITC-BSA) over time from different monophasic CAP nanopowders: hydroxyapatite (HAP), amorphous CAP (ACP), dicalcium phosphate anhydrous (DCPA), and monocalcium phosphate monohydrate (MCPM); (**b**) Release of vancomycin from CAP pastes is controllable by the weight ratio between two different hydroxyapatite components, HAP1 and HAP2, and tunable to anywhere between 0 h and 2 weeks.

Figure 3, however, demonstrates that sustained drug release from pure CAP nanopowders and colloidal pastes is possible. Figure 3a shows four distinct profiles for the release of serum albumin from four CAP nanopowders with different monophasic compositions and different corresponding solubility values [29]. The latter range from 17 g/dm^3^ for monocalcium phosphate monohydrate (MCPM) to 48 mg/dm^3^ for dicalcium phosphate anhydrous, a.k.a. monetite (DCPA) to 0.8 mg/dm^3^ for amorphous calcium phosphate (ACP) to 0.3 mg/dm^3^ for hydroxyapatite (HAP) [30], demonstrating that solubility can be made directly proportional to drug release rates if the phase composition of CAP particles as the carrier is being precisely controlled. The release in this case is, however, driven by undersaturation, not diffusion of the drug outside of the carrier. It is also conditioned by the loading of the drug inside the pores formed by the desiccation-caused compaction of the nanoparticles into a solid form. This implies that the application of such a biomaterial as a bone graft is limited to implantation. Such a release control can also be called tailorable, in a sense that different phase compositions yield different release profiles. However, because no setting of the release properties to any given value within a finite range is possible using one such approach, this form of release control cannot be called tunable as well. Tunability, in the real sense of the word, implies a process of control analogous to the tuning of a radio frequency along a continuous range of frequencies until a desired station playing at a precise frequency is located. To that end, a relationship must be established between the drug release rate and the value of a structural parameter variable within a continuous range. Now, that a truly tunable release from colloidal CAP pastes is possible is shown in Figure 3b. In this case, a complete control over release properties within the range between 0 hours and 14 days is achieved by control over the weight ratio between two differently prepared CAP components in the final mixture [31]. Both components are hydroxyapatite in phase and what they differ in is merely the kinetics of the phase transformation from the as-precipitated amorphous phase to the crystalline final product. Unlike in the case shown in Figure 3a, the release of the antibiotic is driven by diffusion, not dissolution of the carrier, as no considerable degradation of CAP is observed to entail the drug release. Also, in contrast to thoroughly solid CAP from Figure 3a, CAP from Figure 3b is a moldable and cohesive self-setting paste capable of accommodating to the geometry of the bone cavity and solidifying with a similarly tunable kinetics as that typifying the drug release. To that end, it is shown that CAP, albeit a ceramic material, could be made viscous so as to enable the minimally invasive injection into the bone defect site and its thorough filling, without compromising the sustained release of drugs from the material. Most importantly, this means that polymers as viscous components of CAP bone grafts [32,33,34] and components allowing for sustained drug release profiles to be attained might soon be disposed of as excessive.

The two examples shown in Figure 3 demonstrate the versatility of sustained drug release mechanisms achievable using CAP particles as carriers. They also hint at the superfluity of polymers as sustained drug release components of materials for bone regeneration. Eliminating the need for such polymers comes along with multiple benefits, including (a) the reduced cost of preparation; (b) increased scalability; and (c) the solution for the inevitable presence of remnant organic solvent molecules in formulations containing polymers. Namely, in contrast to polymers and lipids, which are precipitated from organic solvents, all CAP nanoparticles reported in this review have been crystallized from aqueous solutions. This gives them an immediate advantage over polymers from the clinical safety perspective [35].

## 3. CAP as an Intracellular Delivery Carrier

**Figure 4:**
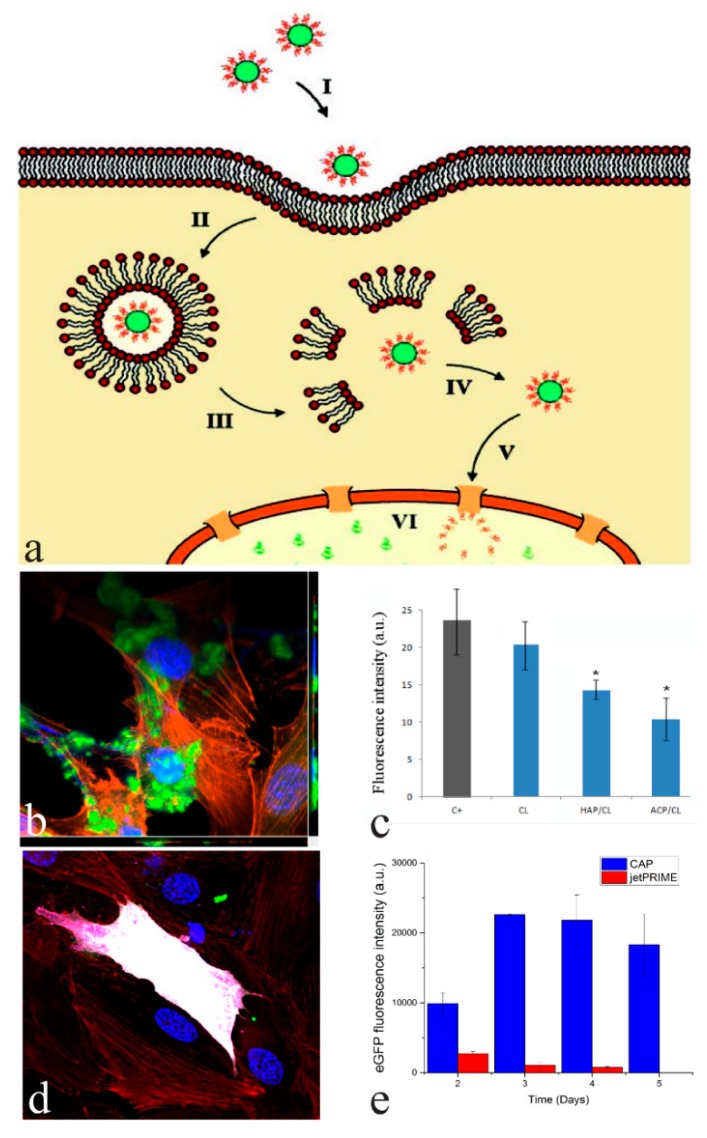
(**a**) Mechanism of the uptake of CAP nanoparticles carrying a nucleic acid (plasmid DNA) into the cell: (I) contact with the cell membrane; (II) uptake via endocytosis; (III,IV) endosomal escape and release into the cytoplasm; (V) a journey toward the nucleus; (VI) nuclear entry and gene expression. Obtained from [45] with permission from ©2008 John Wiley & Sons; (**b**) A confocal optical micrograph showing the uptake of conglomerates of CAP nanoparticles (green) delivering clindamycin inside MC3T3-E1 cells (blue = nucleus, red = f-actin); (**c**) The intensity of fluorescence of intracellular colonies of *S. aureus* co-cultured with osteoblastic MC3T3-E1 cells (C+) statistically insignificantly decreasing following the treatment with pure clindamycin (CL), but significantly decreasing following the treatment with clindamycin-loaded hydroxyapatite (HAP/CL) and amorphous calcium phosphate nanoparticles (ACP/CL). Bars represent averages and values statistically significant (*p* < 0.05) compared to the positive control (C+) are marked with *; (**d**) A confocal optical micrograph showing the transfection of a single MC3T3-E1 cell with a fluorescent protein (purple) in cases when CAP nanoparticles (green) carry a plasmid DNA cargo encoding for the given protein; (**e**) eGFP fluorescence intensity as a measure of the transfection rate in osteoblastic MC3T3-E1 cells transfected using CAP nanoparticles or PolyPlus jetPRIME as the carrier for an identical amount of pDNA encoding for eGFP.

CAP nanoparticles allow for an efficient intracellular delivery of genetic material and other biomolecules [36,37]. For this reason, CAP has been considered a major non-viral transfection agent and a safer alternative to its viral vector counterparts [38]. The mechanism of transfection using CAP nanoparticles, schematized in Figure 4a, is as follows: CAP particles loaded with oligonucleotides are endocytosed via caveolae- or clathrin-mediated pathways [39,40] in a matter of hours following their addition to the medium surrounding the cells [41] (Figure 4b). Travelling down the endosomal route from the membrane toward the nucleus, the endosomes encapsulating CAP particles transition into acidic lysosomes threatening to degrade the nucleic acid cargo. However, CAP begins to partially dissolve already at the comparatively low pH typifying the late endosome, enabling the escape of the nucleic acids before they become cleaved by lysosomal nucleases. This partial dissolution of CAP not only liberates the adsorbed nucleic acids, but also destabilizes the lysosomal membrane by releasing Ca^2+^ ions and blocking the proton pumps thanks to the release of hydroxyl groups. The path of the released nucleic acid from there on depends on its nature: whereas plasmid DNA must make it to the cell nucleus in order to be transcribed, siRNA need not leave the cytoplasm for gene silencing using the means to be achieved [42]. Ideally, plasmid DNA thus travels on its own to the nucleus where it becomes transcribed to mRNA, which subsequently becomes translated to a particular protein structure (Figure 4d). Depending on whether the delivered DNA strand becomes transcribed outside of the genome or becomes incorporated to it, the transfection would be either transient, fading after a few cell division cycles, or permanent. Although CAP nanoparticles can enter the nucleus [43], they are mainly limited to the extranuclear region of the cell. Bioresorbable as they are, CAP particles become degraded down to constitutive ions, which are then included in the metabolic cycles of the tissue or the organism in question. No negative effects on kidneys or other organs are usually observed as a result of the bioresorption of CAP, suggesting that the risks of ectopic calcification following this resorption process and the elevation of calcium and phosphate concentrations in the serum are minimal [44].

CAP nanoparticles could be optimized by controlling the chemical conditions of their co-precipitation with oligonucleotides to surpass commercially available non-viral carriers in terms of transfection efficiency [46]. This is demonstrated in Figure 4e, where the enhanced green fluorescent protein (eGFP) transfection rate achieved using CAP carriers is shown to markedly exceed that promoted by a commercial carrier, Polyplus JetPRIME. Another effect in which CAP outperforms its competitor is the extended period over which the transfection process occurs, all presumably thanks to the slower degradation of CAP nanoparticles inside the cell compared to softer non-viral carriers, such as lipoprotein complexes, phospholipid vesicles or cationic surfactants. Through the control of supersaturation ratio, the precursor solutions mixing rate, pH, ionic strength, concentration of additives, and other synthesis parameters, the size of the particles and, most importantly, their exact agglomeration degree (moderate agglomeration is thought to protect pDNA and facilitate transport across the cell membrane) could be optimized to yield relatively high transfection rates. Further progress in this optimization process might be inextricably tied to the more fundamental understanding of the interaction between oligonucleotides and CAP nanoparticles. The structure of complexes between CAP nanoparticles and nucleic acids (pDNA, siRNA, *etc.*) is, however, still far from being elucidated. One view holds that nucleic acids serve as nucleation surfaces around which CAP nanoparticles crystallize, forming virtual agglomerates held together by the centrally located nucleic acid molecules [47,48]. The alternative view holds that the entrapment of oligonucleotides within the loose agglomerates of CAP nanoparticles is not possible and their physisorption on the particle surface is the only possible scenario. With the further progress in transmission electron microscopy techniques and particularly the ability to focus onto the interface between soft and hard matter, perhaps the fine structure of these complexes will be revealed.

Another type of molecule in need of efficient intracellular delivery using nanoparticles is an antibiotic [49]. Namely, chronic, recurrent osteomyelitis is accompanied by the formation of intracellular bacterial colonies, which are less susceptible to standard antibiotic therapies [50,51]. Shielded from the host immune system, *S. aureus* colonies internalized by the cell provide a reservoir of bacteria that is far more difficultly targeted by the oral or parenteral antibiotic administration routes than bacteria colonizing the bone matrix [52]. To conceive of the right intracellular delivery carrier to eliminate these internal colonies is thus an imperative in designing a perfectly potent anti-infective drug delivery platform. The advantage of CAP nanoparticles is that they can also be utilized to deliver antibiotics intracellularly and reduce the total bacterial population infesting the tissue. The alkaline nature of the most frequently utilized CAP phase, HAP, also prevents the local drops in pH caused by the bacterial colonies and thus minimizes the effect of reduced antibiotic effectivity in the acidic milieu [53]. The effectiveness with which CAP nanoparticles can deliver antibiotics intracellularly is demonstrated in Figure 4c, where a negative control population of osteoblastic, MC3T3-E1 cells infected with fluorescent, FITC-tagged *S. aureus* is posed side by side with the same population of cells treated with clindamycin-loaded CAP nanoparticles. Thanks to the efficient uptake of these particles, the total bacterial number inside the cells drops and the average lifetime of the cells becomes extended [54]. The antibacterial efficiency of antibiotics against an array of multidrug resistant bacteria can thus be increased by a whole order of magnitude when delivered with CAP nanoparticles [55], the reason presumably being the ability of the CAP carrier to penetrate the cell membrane and deliver its load intracellularly.

## 4. CAP as a Foreign Ion Accommodator

One of the essential roles of CAP in the body, alongside serving as a major component of an organ that provides a skeletal support for soft tissues and acts as a factory for the production of blood cells, is to act as a mineral reservoir for the body [56]. In fact, the first evolutionarily formed CAP skeletons presumably had the role of primitive tanks for storage and internal regulation of the concentration of essential microelements. They also had a role to precipitate toxic ions that found their way into the organism and enabled the latter to survive even in highly polluted environments [57]. Thanks to these superior ion-exchange properties, CAP, predominantly in the form of HAP, is being used as an adsorbent in chromatographic columns [58,59] as well as in filters for the removal of toxic elements from contaminated waters [60,61]. This propensity of CAP to engage in ionic substitutions is not even closely matched by other abundant biominerals, such as calcium carbonates, calcium sulfate or silica, let alone metallic particles synthesized by bacteria. It is possible owing to the enormous charge balance flexibility of its crystal lattice and a broad range of Ca/P molar ratios for which the space group of the lattice will be preserved. This makes CAP capable of accommodating more than a half of all the elements of the Periodic Table [62]. In the case of HAP, for example, phosphate groups can be readily substituted with groups such as carbonate (B-type HAP) [63], selenite [64], vanadate [65], silicate [66], and others; calcium ions can be substituted with cations ranging in atomic weight from as light as lithium [67] to as heavy as bismuth [68], thorium [69], and uranium [70]; also hydroxyl groups can be substituted by ions as small as fluoride [71] and as large as carbonate (A-type HAP) [72]. The ease with which these substitutions could be made through simple co-precipitation reactions has led to a large body of research—albeit not very imaginative and somewhat trivial at times—on ion-substituted CAPs and their effects on bioactivity, antimicrobial activity and other biological properties of interest. To this day many ions have been incorporated into HAP in an attempt to impart some of their unique properties to it. Particularly interesting in this context are HAP incorporating magnetic ions (Fe^2+/3+^, Co^2+/3+^, Gd^3+^), photoluminescent ions (Eu^3+^, Yb^3+^, Tb^3+^, Y^3+^), radioactive ions (^125^I^−^, ^99m^Tc), reactive oxide species producing ions (Hf) and ions with pronounced antibacterial (Ag^+^, Zn^2+^, Cu^2+^, Ga^3+^, SeO_3_^2−^), osteoinductive (Mg^2+^, Sr^2+^, CO_3_^2−^, SiO_3_^2−^), angiogenic (Mg^2+^, Si^4+^), antiresorptive (Zn^2+^) and, at times, anticancer properties (SiO_3_^2−^, SeO_3_^2−^). CAPs doped with photoluminescent rare-earth elements provide for a low-cost, resorbable and safer alternative as cell imaging probes to surface plasmonic nanoparticles and semiconductor quantum dots, while retaining a number of advantages that the latter have over organic fluorophores, including higher fluorescence intensity and photostability, broader absorption and narrower emission wavelength ranges, less of the absorption/emission wavelength overlap, and others. For example, Eu^3+^-doped HAP was capable of exhibiting multicolor luminescence under visible light excitation and resistance to quenching up to 15 wt % of Eu^3+^ [73]. Additional substitutions of OH^−^ groups with F^−^ reduce the vibrational modes of the lattice and prevent the quenching of the excited state of the rare-earth ion, facilitating the fluorescent transition [74]. Unlike in the case of quantum dots, however, where the color of the emitted light can be tuned by controlling the particle size, the emission bands can be changed only by changing the chemical identity of the lanthanide dopant. Although the last ionic additives incorporable into CAP will soon be off the list in terms of being integrated into CAP and limitedly characterized for their properties, it will be a while before even the tip of the iceberg of possibilities achievable through the synergy amongst different ionic dopants is revealed. Some of such simultaneous incorporations of two or more types of ions into CAP, such as silver and lanthanides [75], zinc and magnesium [76], selenite and manganese [77], europium and gadolinium [78], have already been tested for their multifunctional properties.

**Figure 5:**
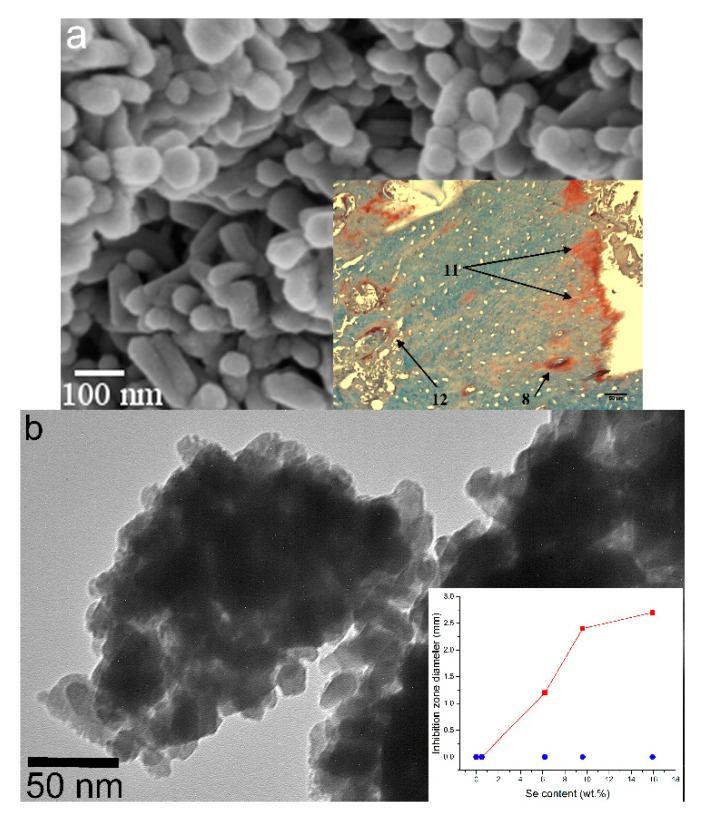
(**a**) SEM image of hydroxyapatite nanoparticles in which Ca^2+^ ions were partially substituted with Co^2+^ ions. The inlet shows an almost complete resorption of the implant and the regeneration of an osteoporotic bone 24 weeks after the implantation (8—formation of Haversian canals; 11—ossification frontline; 12—collagen fibers; all in-between is the region filled by the newly regenerated bone); (**b**) TEM image of selenite-incorporating hydroxyapatite nanoparticles. The inlet shows the diameter of *E. coli* growth inhibition zone around particles of selenite-doped hydroxyapatite as a function of the concentration of selenite ions inside hydroxyapatite particles and depending on whether the selenite ions were incorporated into the lattice by co-precipitation (-■-) or by ion-exchange sorption (-●-).

Figure 5a displays the microstructure of magnetic, cobalt-doped HAP, having the average particle size of 70 nm and exhibiting a more positive effect on regeneration of osteoporotic alveolar bone than its nonmagnetic counterpart, in spite of imposing considerable cytotoxicity on bone cells (not epithelial cells, too, interestingly) *in vitro* [79]. This and other magnetic CAPs are hoped to find an application in anticancer hyperthermia therapies by replacing the more frequently used iron oxide nanoparticles known for their long-term cytotoxicity [80]. Another application would be in magnetic-field-assisted bone regeneration, a therapeutic process whose fundamentals have not been understood yet very well, but whose benefits have been verified in numerous *in vivo* studies [81,82,83]. Figure 5b demonstrates the antibacterial effect of selenite-doped HAP, increasing in direct proportion with the amount of selenite incorporated into the structure of HAP. The choice of selenite is justified by the multifold effect it exerts on biological systems: not only is it antibacterial in nature [84], but it can also exhibit pronounced anticancer properties [85] as well as elicit an osteoinductive response from bone cells [86,87].

## 5. CAP as a Material Formable into Macroporous Constructs for Tissue Engineering

Pores of sufficient size, openness, and interconnectedness in an implant allow the cells to populate its interior and proliferate therein. This pervasion of a biodegradable biomaterial with cells has numerous benefits for the regeneration of a tissue temporarily replaced by its means [88]. Studies on the effect of porosity on the proliferation and the osteoinductive response of cells have indeed shown that macroporous CAP ceramics are favored over their dense, albeit mechanically stronger, counterparts [89,90,91]. These insights have led to the demands for macroporous tissue engineering constructs that are either seeded with cells prior to implantation in the form of scaffolds or acellular and populated with the host cells after their placement in the body [92]. Although polymers have been the traditional material of choice for making such constructs because of their superior flow properties and moldability, CAP nanoparticles could also be formed into tissue engineering constructs with precisely defined porosities using appropriate processing techniques. Freeze drying is a technique that has been proven successful in synthesizing CAP scaffolds, albeit usually involving combinations with a polymeric phase to impart a sufficient mechanical integrity to the scaffold. This technique is often combined with sintering to improve the toughness and the tensile strength of the scaffolds [93]. Anisotropic lamination is observed under certain conditions, resulting in unusually high compressive strengths as well [94], in analogy with the mechanical strengths typifying the anisotropic microstructures of tooth enamel [95] and composite nacre [96]. Another frequently used method for making CAP scaffolds involves the seeding of CAP particles on the surface of a polymeric foam, whose subsequent burning leads to a porous ceramic construct [97]. Particle leaching methods involve the dispersion of particulate additives inside CAP solids or pastes and their leaching upon bringing the material in contact with a solution, leaving pores behind. Scaffolds created from calcium-deficient self-setting HAP pastes via particle leaching, for example, had the interconnectivity of their micro- and macro-pores controllable using the liquid-to-powder ratio [98]. Interestingly, simple setting reactions in the absence of any porogens could also yield porous CAP solids permeable to cells, as exemplified by the 50% porosity of hardened cements whose tunable release properties are shown in Figure 3b.

Thanks to their ability to form viscous pastes, CAP particles could be formed into compact macroporous constructs using computer-aided additive manufacturing and various other stereolithographic and robocasting processing methods. Both HAP [99] and biphasic HAP/TCP [100] compositions were fabricated using 3D printing, the latter of which elicited a more positive tissue integration response. These techniques allow for a far more precise design of the porosity parameters—e.g., the distribution of pore sizes and shapes, the dimension and tortuosity of channels connecting the pores, *etc.*—than the use of particle leaching or freeze drying as processing methods. Consequently, they allow for a more accurate study of the effect of these porosity parameters on the tissue response, the reason for which they are expected to fundamentally revolutionize the field of tissue engineering in general in the near future and solve the enigma as to what the ideal microarchitecture of CAP scaffolds is. Feedback looped with computerized axial tomography, 3D printed CAP scaffolds can also be customized for individual patients and tailored to fit specific bone defects [101]. Such a rapid prototyping setup is an ideal solution for implantable bone graft needs of patients in the clinic.

**Figure 6:**
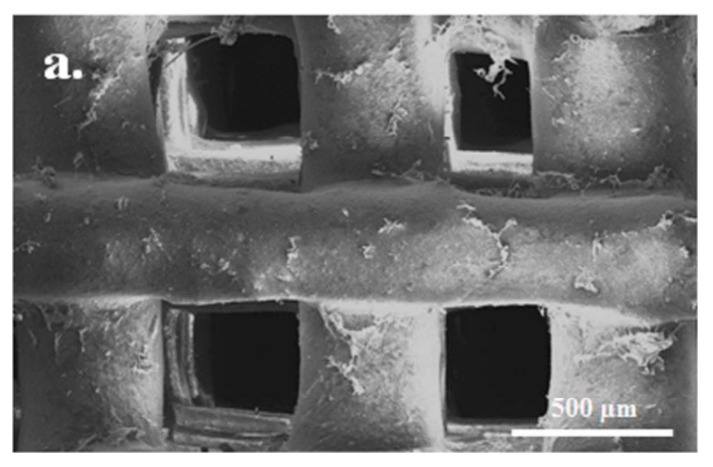

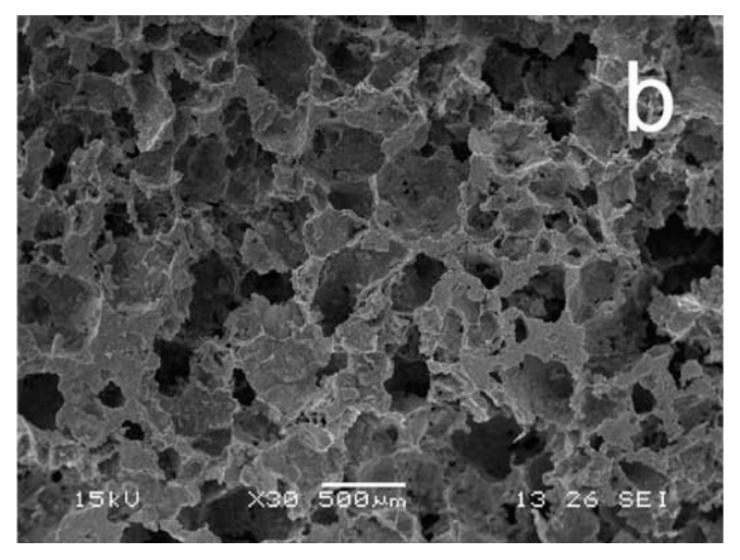
(**a**) A HAP scaffold with highly defined and regular pore sizes, shapes and interconnectivities obtained using 3D printing. Obtained from [109] with permission from ©2016 John Wiley & Sons; (**b**) A HAP scaffold with less controllable pore size, shape and interconnectivity obtained by mixing NaCl with a CAP paste, molding it under a pressure of 2 MPa, then immersing in water to leach out NaCl, and finally vacuum drying to obtain the final, sponge-like scaffold. Obtained from [110] with permission from ©2009 Elsevier.

The question, however, remains as to whether the 3D printed (Figure 6a) or more imperfectly formed CAP scaffolds (Figure 6b) will prove to possess a higher osseo-integrative and osteo-inductive potential. The meaningfulness of this question is supported by multiple findings, including: (a) the ability of irregularly shaped particles to be more therapeutically potent than their monodisperse and perfectly spherical counterparts [102]; (b) the ability of surfaces containing disordered topographic features to promote the differentiation of mesenchymal stem cells to osteoblasts without any chemical factors, unlike their ordered and translationally symmetrical counterparts [103]; (c) the more pronounced osteophilic nature of topographically irregular surfaces than that of their atomically smooth counterparts [104,105,106]; (d) the realization that macroporous scaffolds with rough pore walls induce a greater degree of new bone formation than the same scaffolds with smooth pore walls [107]; and (e) the fact that micropores are equally important in inducing the osteogenic response as the right macroporosity [108]. In agreement with the basic premise of this discourse, which is that the restoration of simplicity is the direction to follow in the design of new CAP biomaterials, it is foreseeable that these sophisticated top-down fabrication methods will eventually give way to the simpler bottom-up syntheses, the reason being the superior cell response and the corresponding cost-to-benefit ratio of the latter. If CAP is an intrinsically imperfect material—cheap, rough, fragile, and lusterless, should not its methods of synthesis adjust to its nature and be equally unpretentious and down-to-earth, that is the question.

## 6. CAP as a Naturally Osteo-Inductive and Osteogenic Material

**Figure 7:**
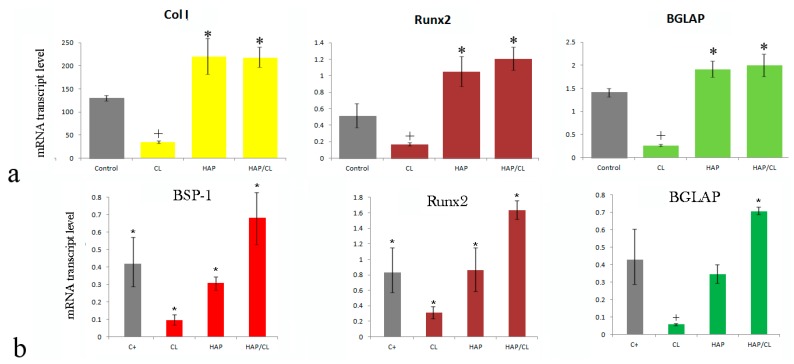
Gene expression of osteocalcin (*BGLAP*), the transcription factor *Runx2*, type I procollagen (*Col I*) and osteopontin (*BSP-1*) in osteoblastic MC3T3-E1 cells uninfected (**a**); or infected (**b**) with *S aureus* and treated with clindamycin (CL), hydroxyapatite nanoparticles (HAP) and hydroxyapatite nanoparticles loaded with clindamycin (HAP/CL). All data are represented as averages and were normalized to the expression of beta-actin (*ACTB*) as the housekeeping gene. Data points significantly different from the control (*p* < 0.05) are topped with an asterisk (*).

CAP has been traditionally termed as an osteoconductive material, as a reference to its ability to promote a viable contact with the boney tissues and become well integrated with them [111]. This traditional view has held that to make CAP osteo-inductive in terms of being able to induce stem cell differentiation into mature bone cells and osteogenic in terms of being able to promote new bone growth, it would have to be supplemented with the appropriate growth factors, e.g., bone morphogenetic proteins and delivered in conjunction with cells, respectively [112]. However, the fact that CAP *per se* can augment the new bone formation and induce the osteogenic differentiation in the absence of any chemical factors is often overlooked. One example comes from Figure 7, where it is seen, first of all, that the administration of clindamycin to osteoblastic MC3T3-E1 cells, be they uninfected with *S. aureus* (Figure 7a) or infected (Figure 7b), downregulates the expression of an array of bone markers, *i.e.*, osteocalcin (*BGLAP*), the transcription factor *Runx2*, type I procollagen (*Col I*), and osteopontin (*BSP-1*). No such downregulation is observed when the same cells are treated with pure HAP nanoparticles. However, after the cells have been treated with HAP loaded with clindamycin, the osteogenic response becomes “rescued”, demonstrating that the delivery of an osteoinhibitory drug such as the antibiotic clindamycin by means of HAP is able to compensate for the negative effects of the drug alone and either restore the osteogenic gene expression to the level of the control sample or in some cases significantly upregulate it with respect to these controls [113]. In another study the addition of HAP to a carboxymethyl cellulose hydrogel promoted the differentiation of dental pulp stem cells to osteoblastic lineage, as evidenced by the upregulated expression of *Runx2*, *Col I*, alkaline phosphatase, osteonectin, dentin matrix acidic phosphoprotein-1 and dentin sialophosphoprotein after 21 days of culture [114]. The expression of osteogenic proteins leptin, leptin-R and Runx2 was also significantly higher in mesenchymal stem cells (MSCs) incubated with HAP or with a biphasic HAP/TCP mixture than in the control group [115]. Osteo-induction involving the upregulation of *BSP* and bone sialoprotein (*BSP-2*) was also detected in MSCs grown on biphasic CAP granules with different HAP/TCP weight ratios. Numerous other studies confirmed the ability of CAP to boost the osteoblastic differentiation, thereby deservedly endowing it with the attribute of osteo-inductive [116,117,118,119]. As far as the osteogenic response is concerned, studies evidencing increased mineralization of cultured cells in the presence of CAP particles [120] and increased bone formation and enhanced bone repair and fusion around implants coated with CAP [121,122] or scaffolds supplemented with CAP [123] can serve as the evidence in its favor.

Not all forms of CAP, however, are able to upregulate the expression of osteogenic markers and boost new bone growth. Understanding the degree to which different particle properties—from size to shape to agglomeration extent to surface charge to topography to microporosity to the exact surface structure to hydration degree and the composition of the Stern layer and beyond—affect this osteogenic response and how they act synergistically is an ongoing effort. For example, it is known that nanosized CAP particles elicit a more intense osteogenic response *in vitro* than their micro-sized counterparts [124,125]. Assessing all these individual properties at the nano scale is made difficult due to the fact that small differences in the nanoparticle size are often more significant than the transition from micro- to nano-sized particles itself. For instance, the melting point of some oxide ceramics changes to a greater degree when the particle size is reduced from 20 to 10 nm than when it is brought down from a few microns to 20 nm [126]. Likewise, nanoparticulate HAP was more toxic to human bronchial epithelial cells when delivered at 50 μg/cm^2^ than when delivered at a twice higher concentration [127]. With hundreds of studies on CAP being published every year, synthetic efforts involving the compilation of data from different studies and deduction of the general findings are today needed more than ever. Still, if CAP nanoparticle properties could be precisely optimized for the most intense osteogenic response, there would be no need for the use of expensive growth factors, as susceptible to degradation and prone to exert a plethora of side effects as they are [128,129,130], which would have tremendous cost-effective repercussions on the development of osteogenic materials in general.

## 7. CAP as a Natural Inhibitor of Osteoclastogenesis

**Figure 8:**
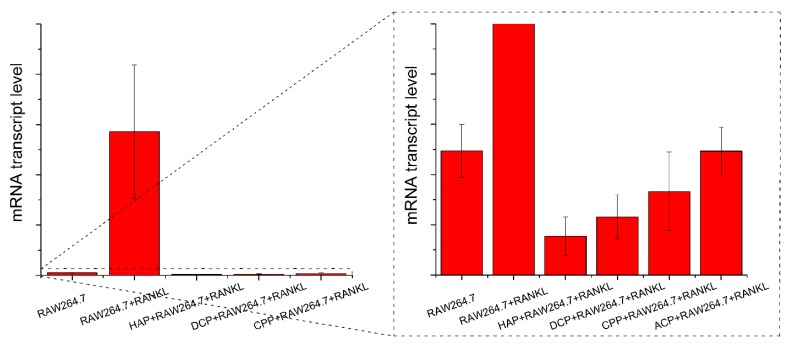
mRNA transcript levels of TRAP as an osteoclastic gene expression marker compared between different cell populations: undifferentiated RAW264.7 cells, RAW264.7 cells differentiated with the use of RANKL, and differentiated RAW264.7 cells subjected to treatments with various types of CAP nanoparticles: hydroxyapatite (HAP), dicalcium phosphate anhydrous (DCP), calcium pyrophosphate (CPP), and amorphous CAP (ACP). Gene expression is normalized to the expression of *POLR2A* as the housekeeping gene. The magnified region of low mRNA transcript levels is shown to differentiate between the effects of different CAP powders.

Bisphosphonates are compounds used to mitigate bone loss due to osteoporosis. This therapeutic outcome they achieve not by reinforcing new bone formation, but by slowing down the activity of osteoclasts [131,132]. Although frequently overseen, CAPs can exhibit the very same effect on osteoclasts. For example, as shown in Figure 8, the addition of RANKL as the promoter of osteoclastogenesis in the RAW264.7 macrophage cell line elevates the mRNA transcript level of TRAP as the osteoclastic gene expression marker. However, the mRNA transcript level of TRAP in cells treated with both RANKL and CAP powders was at a similar level as that in the undifferentiated cells (Figure 8), indicating the inhibitory effect of CAP on the differentiation of RAW264.7 macrophages into osteoclasts. In other words, CAP particles, regardless of their phase composition, are capable of obliterating the effect of RANKL as a differentiation agent when added in combination to RAW264.7 cells. Similar findings were reported in a study by Stražić-Geljić *et al.*: RAW264.7 cells subjected to a simultaneous treatment with RANKL and CAP particles exhibited downregulation of genes such as ACP5 and MMP9 [133]. Inhibition of proliferation, fusion, and TRAP activity of RAW264.7 cells seeded on biphasic CAP ceramics was also reported [134] as well as inhibited differentiation of the same cells into multinucleated osteoclasts upon seeding on a collagen scaffold containing HAP [135]. The complexity of the effects of nanostructured HAP exhibits on osteoclastogenesis is further evident from a study in which HAP did cause a dose-dependent decrease in the osteoclastic gene expression and resorption capacity of mature osteoclasts, but it also had the opposite, augmentative effect on these very same parameters in undifferentiated peripheral blood mononuclear cells [136]. Moreover, as seen from Figure 8, this inhibitory effect exhibited by CAP nanoparticles differs depending on the phase composition of CAP and is most pronounced for HAP, the CAP phase naturally present in bone, and is least pronounced for amorphous CAP. This is in agreement with the previously observed impairment of the differentiation of primary mouse bone marrow macrophages into osteoclasts as a direct function of the weight percentage of HAP in a HAP/TCP biphasic CAP [137]. The TRAP mRNA transcript levels in cells treated with amorphous CAP or calcium pyrophosphate were insignificantly different from that in undifferentiated cells. In contrast, the TRAP mRNA transcript levels in cells treated with HAP or DCP anhydrous were significantly lower than that in undifferentiated cells (*p* < 0.05), suggesting the possibility of tailoring the phase composition of CAP nanoparticles for the desired osteoclastic activity following the surgical insertion of the particles into the body.

## 8. CAP as a Natural Antimicrobial

The rapidly rising resistance of bacteria to antibiotics threatens to produce pandemics of tragic proportions if the solution to this pending disaster is not reached timely [138]. At the moment, the timeline for the passage of new antibiotics from the bench to the bedside is longer than that needed for bacteria to evolve their resistance to it. One approach to solving this problem is based on seeking antimicrobial alternatives to organic molecules susceptible to the issue of bacterial resistance. Metallic nanoparticles [139], bacteriophages [140], and antibacterial peptides [141] have been investigated in search of these alternatives. All of these, however, are associated with considerable health risks for the patient. Silver nanoparticles, for example, more researched for this application than any other inorganic systems, are heavy metals and are, as such, hazardous by default. In fact, size-dependent cytotoxicity of silver nanoparticles has been frequently observed *in vitro* [142,143,144]. As is the case with all viral entities, the use of bacteriophages entails a finite immunological risk for the patient, even though they are not human-specific [145]. They are also limited in terms of their ability to infect only a small number of genetically different bacterial strains, which frequently renders them helpless against many chronic infections, including osteomyelitis, wherein tissues become colonized by multiple genetic variants of single or multiple bacterial species. Finally, antimicrobial peptides are costly to synthesize, store and combine with the right carrier, alongside being broken down in the body relatively fast and carrying a permanent risk of denaturing in the body and causing an inflammatory response. All in all, in spite of their broad activity and low resistance potential, their potential immunogenicity, poor bioavailability and high production costs have been stumbling blocks on the road to their transformation into a major anti-infective drug type [146].

**Figure 9:**
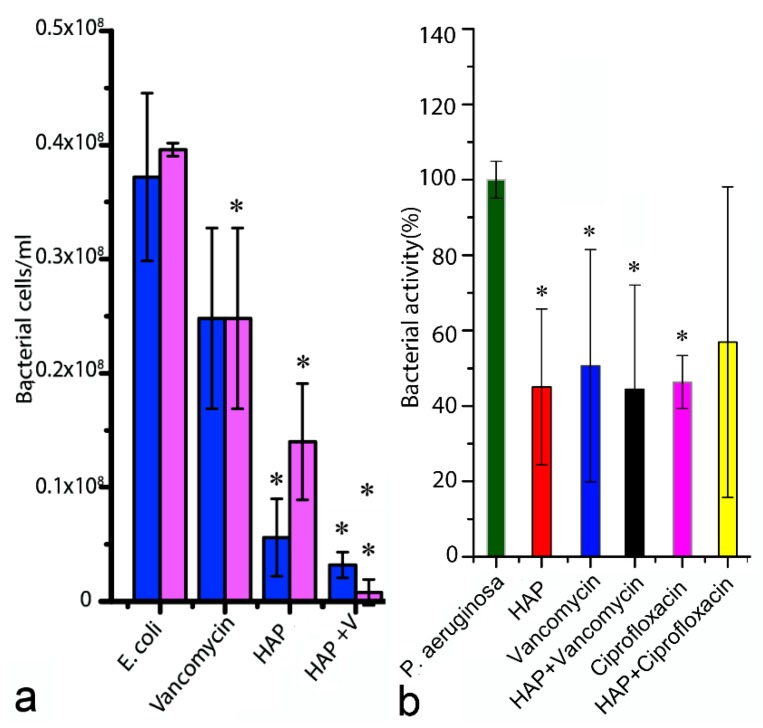
A difference in (**a**) the number of *E. coli* bacteria per cm^3^ in LH broths; and (**b**) bacterial viability in the biofilm of *P. aeruginosa* following overnight incubation with pure HAP pastes, with pure antibiotics (ciprofloxacin or vancomycin) or with HAP pastes loaded with antibiotics. Data points significantly different from the untreated controls (*E. coli*, *P. aeruginosa*, *p* < 0.05) are topped with an asterisk (*).

Another inherent weakness of traditional antibiotics comes from the nature of the mechanism of their action: namely, their bactericidal and bacteriostatic effect is conditioned by the physical contact of the drug with the cell membrane. In the case of osteomyelitis, however, a large percentage of the bacterial population resides in the biofilm [147]. These cells are comparatively quiescent and buried underneath a thick sheet of an exopolymeric substance that repels microbicides and makes the cells largely inaccessible to antibiotics [148,149]. In addition, biofilms are powerful mediators of the horizontal resistant gene transfer and of quorum sensing, the coordinated gene regulation response by a bacterial population to an invader, making the cells inhabiting them also more intrinsically resistant to antibiotics than the planktonic cells. An ideal antibacterial therapeutic is thus expected to satisfy two essential prerequisites: (a) not to cause the target to develop resistance to the therapy; and (b) not to harm the host. Neither the traditional antibiotics nor any of the aforementioned alternatives satisfy these two essential prerequisites. Antimicrobial particles that could stay in the body for prolonged periods of time without causing the inflammatory reaction or a cytotoxic response are needed and CAP, a natural biomaterial, would be an excellent choice if it only had an antibacterial activity. Interestingly, although CAPs usually do not possess any detectable antimicrobial properties, they could be designed to possess them. For example, in a surface-active, nanoparticulate form, the antibacterial activity of CAP can be comparable in strength to that of antibiotics such as vancomycin or ciprofloxacin and is particularly pronounced against gram-negative bacteria, such as *E. coli*. This is demonstrated in Figure 9a, where the number of bacterial colonies per volume of a broth following overnight incubation with pure CAP pastes is comparable to or lower than that in broths incubated with either CAP pastes loaded with antibiotics or antibiotics alone. Pure CAP is also extraordinarily effective against *P. aeruginosa* biofilm, as demonstrated in Figure 9b. The mechanism for these unusual antimicrobial effects is not clear yet, though it most probably stems either from surface chemistry changes accompanying the amorphous-to-crystalline phase transitions involved in solidifying CAP pastes or from the release of ultrafine CAP particles that block specific pathways or compartments in bacteria and cause their death. Therefore, although CAP allows for the sustained release of antibiotics, it can also substitute for their action and, thus, technically, eliminate the need for them. Finally, unlike the aforementioned alternatives to traditional antibiotics, CAP is a nontoxic, bioresorbable material, naturally present in the body, and is far less likely to cause any considerable health risks to the patients. Our recent realization of the antibacterial properties possessed by the systematically optimized CAP nanoparticles^31^ opens a new avenue in their research, extending far from the realm of bone regeneration and into the broader area of anti-infective therapies and wound healing. If CAP as a bioactive coating on load-bearing metallic implants in orthopedics could simultaneously endow the implant surface with antifouling properties, it would present a major step forward in the design of advanced materials for bone regeneration. This step would proceed in the direction of which bone engineers could have only dreamt: that is, how to create a surface that is bioactive, yet antifouling, a surface that is welcoming for bone cells, yet repellant for bacteria? With CAP nanoparticles being increasingly used as intravenous vehicles for the controlled, typically pH-responsive delivery of proteins [150,151,152], small molecules [153,154,155], and calcium ions [156], entering the area of antimicrobials for prophylactic or remedial purposes would be another step in the progression toward expanding the repertoire of their applications in the biomedical arena.

## 9. CAP as the Ugly Duckling Concealing a Swan Within

The sketching of the way for the future research in CAP performed here, undoubtedly promising, can take on an almost fairytale character if we recall the plentiful of deficiencies that characterize this material. For example, the hydrated and diffusive surface layer of ions prone to engage in constant restructuring via dissolution/reprecipitation prevents the stable chemical conjugation of CAP with therapeutic ligands and puts it, in that sense, behind materials such as silica, gold or carbon nanotubes, let alone organic structures. Annealing could somewhat stabilize this surface layer by increasing the crystallinity and lowering hydration; however, avoiding sintering, colloidal destabilization, uncontrollable particle growth beyond the nanometer range, and reduced bioactivity is an accompanying challenge.

Because of its characteristic growth proceeding through aggregation of Posner’s clusters and larger particulate units, the control over particle size and morphology is challenging, to say the least. As usual, however, the cause of a problem can be a key to its solution; in this case, this aggregational growth endows CAP with a characteristically low crystal growth rate, involving the orientational recognition of coalescing crystals through successive contact and dissolution [157], thus bearing more resemblance to the growth of protein and viral crystals than of ordinary inorganic salts [158]. The effort to take advantage of this slow crystal growth to build attractive morphologies and architectures on the nano scale using organic, biomimetic additives is, however, at its earliest beginnings in spite of the decades of attempts to grow biominerals in the lab setting.

Then, although the solubility product is indeed a determinant of bioresorbability when it comes to calcium orthophosphates [159], degradation of CAPs *in vivo*—like that of most ceramics except the biosoluble ones—and the consequent release of the payload take place through cell/material interaction (lactic acid release, phagocytosis, *etc.*) rather than through hydrolysis and/or physical, porosity-controlled release as in the case of polymers. This implies that in spite of the ability of CAPs to incorporate a plethora of therapeutically effective ionic species, their release is difficult to predict, largely depending on the biological makeup of the area in the body in which CAP is being administered, which is subject to large variations. Reflected in the highly complex and variable formation and phase transition pathways, the compositional and structural volatility of CAPs is often considered as another demerit of theirs, even though the potentials of its harnessing for various therapeutic or diagnostic effects have been barely tapped into.

The low zeta potential of CAP particles, typically <±15 mV in the entire pH range of stability [160], is conducive to their aggregation, the reason for which their preparation in a lastingly stable colloidal form needed for many gene and drug delivery applications is near to impossible without the use of proper additives. This moderate instability of CAP dispersions can, however, be an advantage for transfection, especially in 2D culture, where particle sedimentation is a prerequisite for cell/particle contact to occur. Another propensity that should be in favor of CAPs as transfection agents is that toward the attraction of an abundant and compositionally versatile protein corona, which, as it has been shown [161], favors the uptake by the cells; though not measured in serum or cell culture media yet, the lipoprotein corona around HAP particles passing through the pulmonary surfactant monolayer was richer and, thus, more intrusive than that around hydrophobic polystyrene particles [162], being the natural corollary of the excellent adsorption properties of CAP.

The fact that the atherosclerotic plaque is largely composed of HAP crystals [163,164], which engage in epitaxial co-precipitation with cholesterol [165], presents another major concern before the use of CAP nanoparticles as intravenously deliverable drug carriers. Fine tuning of the particle structure and composition is, however, capable of overcoming this issue whose risks are minimal in view of the fact that the serum is supersaturated with respect to CAP and that in a healthy organism the intravenous delivery of CAP should not present an issue at low to moderate dosages. As a result, CAP nanoparticles were safely delivered even at relatively high dosages of 300 mg/kg of body weight [166] and were used for selective organ targeting even outside the reticuloendothelial system [167].

Though injectable, CAP pastes suffer from frequent filter-pressing problems caused by the different flow rates of particles and the liquid medium under the extrusion pressure, resulting in solid phase segregation and the clogging of the syringe [168]. On the other hand, they leave a large room for the optimization of the flow and cohesion properties through the control over particle size distribution, solid-to-liquid ratio, ionic strength, and viscosity of the liquid phase and other parameters, thanks to which these issues could be overcome. The time sensitivity of their clinical application, normally demanding *in situ* preparation just prior to the injection, presents another problem, mostly of regulatory nature, tied to the use of injectable CAPs.

Last but certainly not least, problematic mechanical properties [169], including brittleness, low fatigue resistance, low Weibull modulus, low fracture toughness, and low tensile and bending strengths present another major limitation to the use of CAP in load-bearing implants in orthopedics and dentistry. The tooth enamel, however, albeit not synthesizable *in vitro* as of today [170], has served as a paradigmatic example of the ways in which the resistance to crack propagation, *i.e.*, fracture toughness, could be improved in the absence of any additives: through hierarchical ordering of CAP particles with distinct symmetries at different spatial scales. In other words, many of the intrinsic weaknesses of CAP have the potential to be overcome thanks to its structural, compositional, and interactive versatility. After all, there may be a profounder analogy derivable from the fact that the choice for the main ingredient of the skeleton of our bodies fell in the course of evolution on none other but this material than the usual reference to its sparsely soluble nature that endows CAP with the optimal remodeling rate and allows it to act as a mineral reservoir *in vivo*. In short, it may suggest that the foundations for the design of versatilely applicable nanostructures in biomedicine should similarly be built out of CAPs.

## 10. Conclusions

Designing therapeutic nanoparticles for use in tissue engineering and drug delivery, while taking regional social practices and limitations into account, is a rare concept among biomedical scientists. It is, however, a concept that reconnects with E. F. Schumacher’s ideal also known as “small is beautiful” [171], according to which the transfer of technologies from rich to poor populations must be accompanied by the modification of these very same technologies in terms of their size, scale and other characteristics, all for the purpose of their matching the limitations within which the local communities thrive. Nanotechnologies are by no means an exception to this necessity to understand the context of the local culture to which the new technology is exported and adjusting it accordingly in order to ensure its congruous adoption and contribution to sustainable development [172]. With the science of nanoparticle synthesis, characterization, and functionalization often being termed “science of the small”, these two emphases on “small”, one of which is scientific in nature and the other one of which is societal, should perhaps be pursued in parallel more often than they are.

In this review we have shown that designing a nanoparticle for such a socially responsible tissue engineering application requires going against the stream defined by the current trends in the evolution of scientific thought in the field of nanoscience and nanotechnologies. In particular, while there is no doubt that the future in the realm of nanoparticle synthesis belongs to the further complexification of its structure and properties, this review serves the purpose of demonstrating that sometimes it may pay off to move in the opposite direction, that is, against the mainstream and against the trends imposed by the scientific community. In other words, to simplify the nanoparticle structure might lead to tremendous social benefits. Specifically, we have shown that CAP is a multifaceted material that conceals an array of utilizable latent properties. Revealing them is especially fulfilling given the history of deficiencies that epitomize CAPs and that limit their use as biomaterials, including brittleness, low fracture toughness, incapacity for stable chemical conjugation, a formidable processing potential compared to metals, non-oxide ceramics or polymers, *etc*. Though CAP has been traditionally considered a “poor man’s material” among tissue engineers, an extraordinary richness of properties hidden underneath this ostensible poverty is being increasingly revealed, the continued elicitation of which and consideration for use in biomedicine irresistibly echoes Mother Teresa’s celebration of poverty in every aspect of living.

In this review we call for the partial reversal of the direction of research in CAP, from complexification to simplification, finding in the versatility of properties dormant in this material the route for its promotion into a key material for socially responsible tissue engineering. Further investigation of this intrinsic proteanism may bring us closer to an affordable-to-all bone replacement material containing multifunctional CAP as a sole component. Although we foresaw in this study CAP as a potential compensator for the role of antibiotics, osteogenic growth factors, antiresorptive bisphosphonates, radiographic and contrast agent molecules, optically active compounds, polymers as viscous components, polymers as additives enabling controlled drug release, and multiple other exquisite components of tissue engineering constructs, we do not wish to trivialize or discourage future research on supplementation of CAP with all these different components. Rather, what we call for is a complementation of this indisputably valuable line of research whose aim is further complexification of the biomaterial structure with a reverse path, whose aim is continued exposition of medically applicable properties dormant in CAP alone. Such a fundamental line of research may simultaneously bring us closer to a practical, therapeutic option that would be utterly inexpensive, *in situ* synthesizable and affordable to practically every region of the world, from the wealthiest to the poorest. To that end, one such research would not only be guided with the socially aware eye focused on the whole, but would also contribute to the healing of the gap between rich and poor, the gap whose impassability presents one of the most critical threats for the sustainability of the Earth as we know it.

## Figures and Tables

**Table 1:** The prices for commercially available chemical components of advanced bone graft formulations, listed together with companies producing them. By far the cheapest of them calcium phosphates (CAPs), are colored in red. Prices are given for raw compounds and do not include the cost of processing into forms tailored for specific medical applications, e.g., microspheres, macroporous constructs, *etc.*

Class of Chemical	Chemical	Company	Price per Gram
**Antibiotic**	Gentamycin	Thermo Fisher	$110
	Vancomycin	Sigma Aldrich	$51.50
	Ciprofloxacin	Alfa Aesar	$11.10
**Growth factor**	IGF-1	Sigma Aldrich	$4,540,000
	TGF-β 1	Alfa Aesar	$204,000,000
	BMP-2	Thermo Fisher	$12,000,000
	BMP-4	Thermo Fisher	$19,000,000
	BMP-7	Thermo Fisher	$19,000,000
**Bisphosphonate**	Alendronic acid	Sigma Aldrich	$1,690
	Zoledronic acid	Sigma Aldrich	$466.70
	Pamidronic acid	Sigma Aldrich	$4812
**Mineral compound**	Calcium phosphate	Sigma Aldrich	$0.12
	Calcium phosphate	Alfa Aesar	$0.06
**Drug release polymer**	PLGA	Sigma Aldrich	$67.40
	PEO	Alfa Aesar	$0.71
	PLLA	Sigma Aldrich	$45.00
**Viscous polymer**	Polyurethane injectables	Sigma Aldrich	$23.50
	PCL	Sigma Aldrich	$8.74
	Chitosan	Alfa Aesar	$0.84
	Hyaluronic acid	Sigma Aldrich	$26,700
**Luminescent dye**	Oxytetracycline	Sigma Aldrich	$1,632
	Calcein green	Thermo Fisher	$313,000
	Alizarin red	Sigma Aldrich	$2.40
	Xylenol orange	Sigma Aldrich	$41.00
